# The importance of the posterior joint space for functional mandibular movements: A laboratory cross-sectional study

**DOI:** 10.4317/jced.54168

**Published:** 2018-01-01

**Authors:** Cláudia-da Costa Cordeiro, Daniel-Humberto Pozza, Tadachi Tamaki, Antônio-Sérgio Guimarães

**Affiliations:** 1 DDS, MSc in Morphology, UNIFESP/Paulista School of Medicine, Brazil; 2 DDS, PhD, Associate Professor, Department of Biomedicine, Faculty of Medicine and Faculty of Nutrition and Food Science, Universidade do Porto, I3s, Porto, Portugal and Universidad Europea de Madrid; 3 DDS, PhD, Deceased 12 August 2014. Professor Emeritus, Department of Dentistry, Universidade de São Paulo, São Paulo, Brazil; 4 DDS, PhD, Associate Professor, Pain Experimental Laboratory, Faculdade São Leopoldo Mandic, Campinas, Brazil

## Abstract

**Background:**

The search for the ideal, healthy and reproducible position of the condyles is of utmost importance for dental diagnosis and treatment. Thus, the objective of this laboratory cross-sectional study was to verify the relationship between the posterior joint space and the mandibular lateral movements.

**Material and Methods:**

Dental casts from 15 women and 15 men with normal mastication, 28 natural teeth and no history of temporomandibular disorders or pain, were fabricated and mounted on a fully adjustable articulator. From the maximum intercuspal position, condylar displacement was evaluated and measured on the working and nonworking sides during mandibular lateral movement, both to the right and left sides.

**Results:**

The correlation between the measures of interest was assessed with the Pearson correlation coefficient (α=.05). Condylar displacement on the working side and nonworking side condyle was 0.88±0.71 mm and 3.57±1.11 mm (right mandibular lateral movement); and 0.91±0.58 mm and 3.51±0.78mm (left mandibular lateral movement), respectively. No significant correlation in the condylar displacement between the working side condyles on the right and on the left sides was observed (r=.22; P=.248). The condylar poles of the articulator moved posteriorly, simulating the functional movements of the mandible during mastication. In all cases, condylar displacement during mandibular lateral movement both to the right and left occurred posteriorly on the working side condyle.

**Conclusions:**

The condylar poles of the articulator moved posteriorly simulating the functional movements of the mandible during mastication. Moreover, left and right working condyles may require slightly different spaces to function, suggesting minor anatomical asymmetries.

** Key words:**Mastication, dental occlusion, prosthodontics.

## Introduction

The temporomandibular joint movements are controlled by the morphology of the synovial joint and also by the dentition with teeth being the hard end point of closure. Thus, occlusion may influence its function (1). The disk and the condyle are closer to the temporal joint eminence, favoring the existence of a posterior space inside the joint cavity filled by soft tissues (2).

The mandibular sideway movements, in which the condyles slide along the mandibular fossa, has been named the “Bennett Movement” (3). Nearly fifty years after, Sicher (4) demonstrated that the working side condyle not only rotated but also moved gently to side, backwards and inward, while the nonworking side condyle moved down and forward. Therefore, the working side condyle in addition to rotating, also moves gently to the posterior space inside the joint cavity, retracting from 0.5 to 1 mm (5-7). This terminal position of the intercondylar rotation axis, which is achieved when the mandible is in a retrusive position, might be a desirable starting point in case there is a need for extensive restorative reconstruction, and it could be used as a reference point for the intermaxillary relationship. However, this may not necessarily be the ideal physiological functional position (6-9).

Mastication is initiated and finalized with the dental arches in maximum intercuspal position, also called habitual occlusion (HO). By force of this physiological reflex, individuals tend to always close the jaw in HO from any position taken previously. The same is also true when teeth have been assembled or reconstructed in centric relation. Because the contraction of the muscles of mastication projects the jaw to HO, dental relationship in the centric relation may not be a stable position (9-11). Thus, the repositioning of the dental occlusion posteriorly does not seem to be part of the usual masticatory movements. Therefore, the path taken within the joint cavity by the condyles during the mastication has a direct relationship with occlusal inclined planes, indicating that the masticatory pattern is influenced by occlusion (1,9,12). Moreover, it is also known that the sensorimotor cortex may be affected by the location of the occlusion of the prosthesis, leading to the disruption of functional activities such as mastication and functional movements of the maxillofacial area (13).

The fully adjustable articulator Tadachi Tamaki is an adaptive device in the Weinberg rating scale (4) that has the ability to displace the condylar poles in the anterior-posterior direction, allowing the recording of facial asymmetry, and also to take the assembled dental cast to another occlusal position, without the need to be reassembled. The condylar displacement of the articulator corresponds to the movement that the condyles perform inside the glenoid fossa in relation to the occlusal position (14).

Therefore, the ideal position of the condyles, considered to be healthy and reproducible for dental diagnosis and treatment planning needs to be further examined (8,15). In order to reduce uncertainties and to verify the relationship between the posterior joint space with the functional mandibular movements, the aim of this laboratory cross-sectional study by indirect method was to measure and evaluate condylar displacement relative to the glenoid fossa during mandibular lateral movement by using a fully adjustable articulator.

## Material and Methods

Dental casts of 30 patients were mounted on a fully adjustable articulator and condylar displacement was measured during mandibular lateral movement. This laboratory study was approved by the Ethics Committee of the Federal University of São Paulo - UNIFESP, Brazil (protocol Nº 1085/02). All participating individuals signed an informed consent.

Thirty young individuals equally distributed by sex (15 males and 15 females), matched for age (20-36 years), with natural dentition (28 teeth), presenting no periodontal disease, no crossbite, no diastema, no anterior open bite, and no history of symptoms or changes during mandibular movement, were selected for the study. Impressions of the upper and lower arches of each individual were taken, always in the same position, with impression trays filled with irreversible hydrocolloid, according to manufacturer’s instructions. The impression trays were then filled with type IV dental stone up to the cervical level of all dental elements, and type III dental stone in the complementary layer.

The study was performed with the use of the indirect method. Dental casts were mounted on a Tadachi Tamaki fully adjustable articulator. All steps from transferring the facial arch to the articulator, the assembly of dental casts, as well as the observation of the direction of condylar displacement, measurements and data collection were conducted according to the recommendations of the manufacturer by a single evaluator, who was previously trained and calibrated by the creator of the articulator.

**Figure 1:**
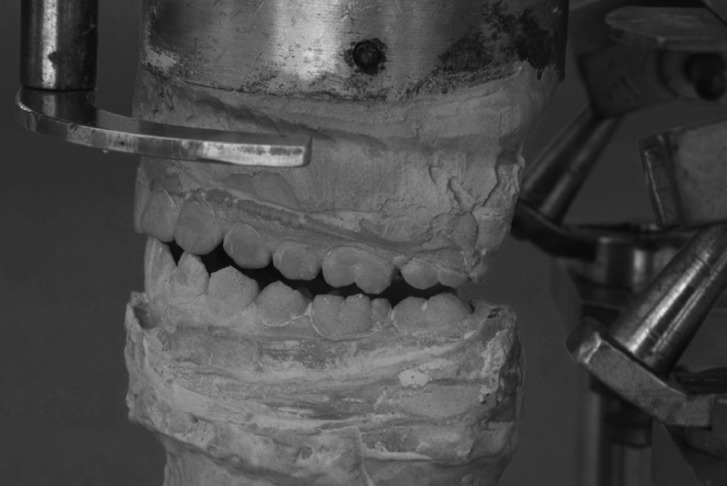
Mandibular lateral movement to right and left sides to canine guidance.

**Figure 2:**
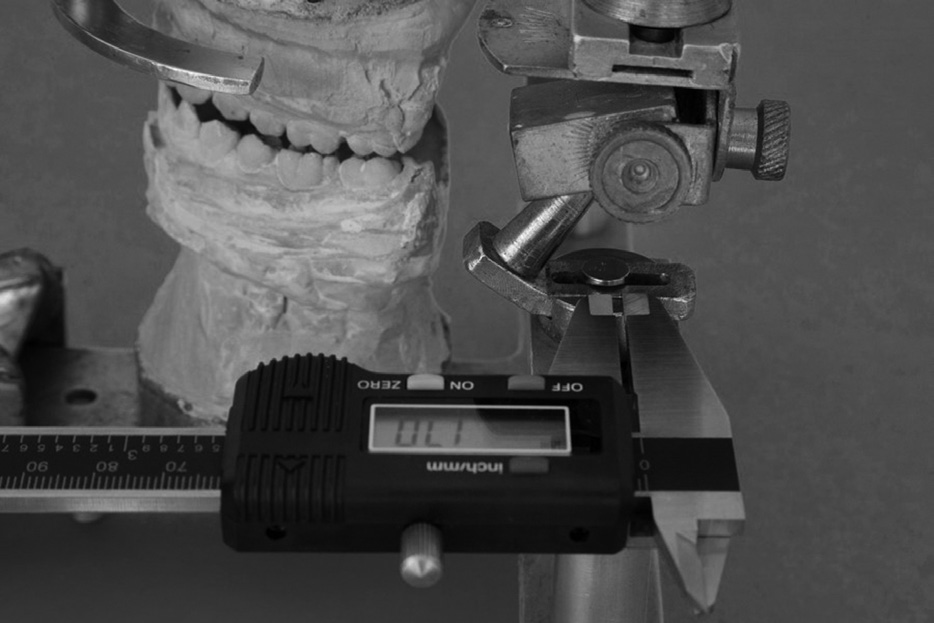
Measurement of condylar displacement with digital caliper.

Dental casts were assembled on the articulator in maximum intercuspal position. From this position, the bolts were loosened, allowing the condylar poles to move right and left. Mandibular lateral movements to the right and left were performed to the canine guidance position, i.e., the contact point of the cusps of the upper and lower canines (Fig. 1). The articulator bolts on the working and nonworking condyle side were tightened to maintain the position, and condylar displacement on the working and nonworking sides were measured with a digital caliper, on both the right and left sides (Fig. 2). During movement of the working and nonworking condyles, the articulator moves in the opposite direction to that in humans, not interfering with the measurements.

For the statistical analysis, the quantitative variables were represented in terms of means, standard deviation (SD), minimum and maximum values; while the qualitative variables were represented in terms of absolute (n) and relative (%) frequencies. The correlation between the measures of interest was assessed with Pearson’s correlation coefficient at a level of significance of α=5%.

## Results

**Figure 3:**
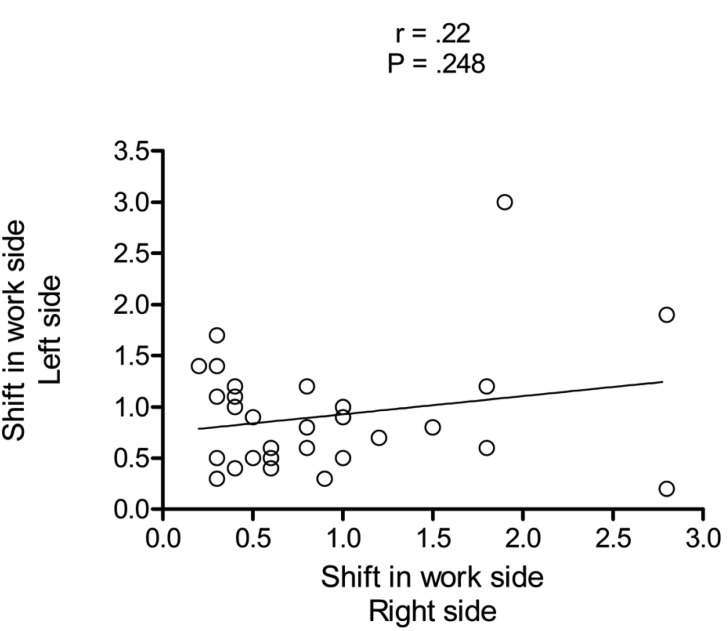
Graph showing the distribution of condylar displacement of working side condyles from maximum intercuspal position during mandibular lateral movement to the right and left side.

In all 30 cases (100%), condylar displacement during mandibular lateral movement from the maximum intercuspal position, to the right and left on the working sides occurred posteriorly. Displacement of the working and nonworking condylar poles on both sides are presented in  Table 1. No statistical correlation between the displacement of the right and left working side condylar poles was found (r=0.22; *P*=.248), as illustrated in figure 3.

**Table 1:**
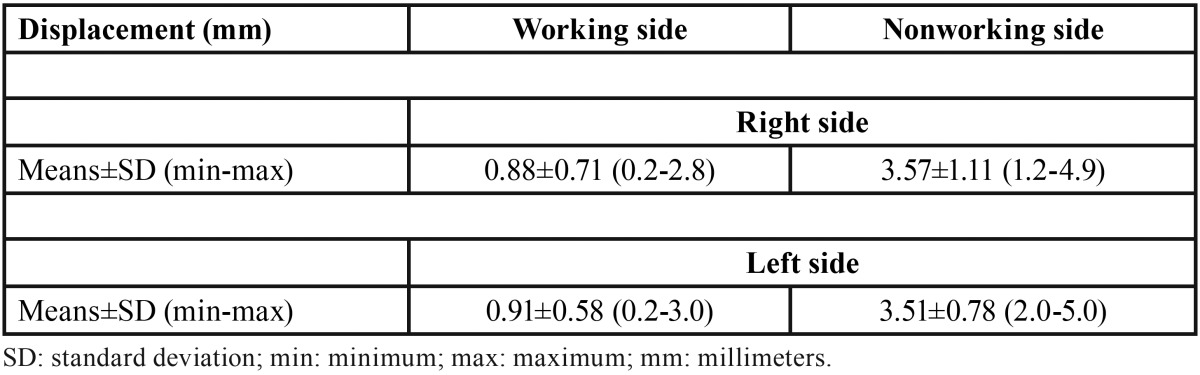
Displacement of the working and nonworking side condyle during mandibular lateral movement from maximal intercuspal position to the right and left sides (n = 30).

## Discussion

The results of this laboratory study demonstrated that the condylar poles were displaced posteriorly during mandibular lateral movement, using the virtual posterior joint space, both on the working right and left sides of the jaw. The displacement of the working side condyles on the right and left sides demonstrated no significant correlation.

Although there are authors who still conduct the measurement of the joint spaces without relating them to the movement of the condyles, the use of dental casts mounted on articulators has been recommended to evaluate the position of the condyle in relation to the occlusion and the glenoid fossa (14,16). Thus, in this study, the measurements were performed, permitting to conjecture the condylar position within the glenoid fossa during mastication. It was found that the displacement of the condyles of the articulator corresponded to the motion effected by the condyles during mastication (14,17).

Mastication involves complex movements of the jaw, not only vertically but also laterally and anteroposteriorly, in which the working side condyle moves backwards. The ability to retract the jaw from the maximum intercuspal position is one of the signs of a well-balanced masticatory system (5-9,18,19). Although the masticatory movements can be altered by changes in occlusion, dental interventions should be consistent with functional movement patterns. Therefore, condyles move within the glenoid fossa following mastication in accordance to the occlusal inclined planes, indicating that the masticatory pattern is determined by occlusion (12,20,21). From the maximum intercuspal position, a reduction of the posterior condylar space was observed as a result of the reproduced mandibular lateral movements. The results of this study are also similar to those found by Gibbs (5) and Kubein (22), who showed that during mastication the condyles perform one degree of freedom anteroposterior movements; being smaller on the working side condyle when compared to the nonworking side condyle.

Although, in some subjects with lateral malocclusions, the size of the condyles may differ substantially, these differences do not generally interfere with TMJ function, demonstrating its plasticity (23). The mandibular condyles, which are part of a single bone, have unique characteristics and are functionally dependent (1,9,10). Thus, as expected in this study, no significant differences between the right and left working sides were observed with respect to the displacement of the condyles from the maximum intercuspal position during mandibular lateral movement. Furthermore, we found that the Bennett movement occurred on both sides in all cases.

Despite the “normal” position of the condyles has not been clearly defined, in the past there were studies that proposed therapies for their repositioning (24,25). As a result, an increased number of therapeutic procedures to “normalize” the position of the condyles has been observed (24,26). Considering the importance of the posterior space to the condyles for the physiological functioning of the jaw during mastication, it was previously observed that the centric occlusion position is occlusally less stable than the maximum intercuspal position (11,15). Thus, to force mandibular positioning to centric occlusion generates instability, which can be followed by a compensatory return to the maximum intercuspal position, or even promote overloading of the TMJ (6,8,11). Due to the great variability of the position, form and size of the condyles, the most favorable, healthy and normal position for diagnosis and treatment planning, should be the one that respects the physiologic needs (9).

Considering the small number of published studies, as well as the lack of strong evidence on the best actions on the TMJ during oral rehabilitation, translational studies followed by randomized controlled trials are required to assess the consequences of different rehabilitation occlusion possibilities, such as the mandibular positioning in centric relation or maximum intercuspal position.

## Conclusions

The present study demonstrated that the condylar poles of the articulator moved posteriorly simulating the functional movements of the mandible during mastication. Moreover, the left and right working condyles may require slightly different spaces to function, suggesting minor anatomical asymmetries. Finally, a correlation between the movements performed by the working and nonworking side condyles, both to the right and left side, was not observed.
